# Precision nicotine metabolism-informed care for smoking cessation in Crohn’s disease: A pilot study

**DOI:** 10.1371/journal.pone.0230656

**Published:** 2020-03-26

**Authors:** Elizabeth A. Scoville, Hilary A. Tindle, Quinn S. Wells, Shannon C. Peyton, Shelly Gurwara, Stephanie O. Pointer, Sara N. Horst, David A. Schwartz, Dawn W. Adams, Matthew S. Freiberg, Vanessa Gatskie, Stephen King, Lesa R. Abney, Dawn B. Beaulieu

**Affiliations:** 1 Division of Gastroenterology, Department of Medicine, Vanderbilt University Medical Center, Nashville, Tennessee, United States of America; 2 Department of Medicine, Vanderbilt University Medical Center, Nashville, Tennessee, United States of America; 3 Geriatric Research Education and Clinical Centers (GRECC), Veterans Affairs Tennessee Valley Healthcare System, Nashville, Tennessee, United States of America; 4 Division of Cardiology, Department of Medicine, Vanderbilt University Medical Center, Nashville, Tennessee, United States of America; 5 Division of Gastroenterology, Wake Forest School of Medicine, Winston-Salem, North Carolina, United States of America; 6 Division of Gastroenterology, The Ohio State University Wexner Medical Center, Columbus, Ohio, United States of America; 7 Department of Pediatrics, Vanderbilt University Medical Center, Nashville, Tennessee, United States of America; Keele University, UNITED KINGDOM

## Abstract

**Introduction:**

Smoking is a strong risk factor for disease severity in Crohn’s disease (CD) and cessation improves outcomes. The nicotine metabolite ratio (NMR) predicts cessation success with pharmacotherapy: varenicline doubles cessation over nicotine replacement therapy (NRT) for “normal”, but not “slow” metabolizers. Varenicline side effects are heightened in slow metabolizers. Methods using NMR to optimize cessation pharmacotherapy have not been evaluated in CD.

**Aims:**

We aim to determine the prevalence of smoking in a CD population and then assess these smokers’ attitudes toward a personalized metabolism-informed care (MIC) approach to cessation.

**Methods:**

In this observational study, we surveyed 1098 patients visiting an inflammatory bowel disease center about their smoking history. We then evaluated a subgroup of individuals with CD (n = 32) who participated in a randomized controlled trial of smoking cessation using MIC versus usual care. For MIC, medication selection was informed by the NMR (normal ≥0.31 vs. slow <0.31). The primary outcomes were intervention satisfaction and match rates between NMR and medication choice.

**Results:**

The baseline prevalence of smoking in our CD population was 13%. Intervention participants reported high rates of satisfaction (85%) and chose a medication that matched their NMR result more often in the MIC group (100% vs. 64%, p = 0.01). Six of 16 (37.5%) patients prescribed varenicline discontinued due to side effects.

**Conclusion:**

MIC produced high rates of satisfaction and matching between NMR and medication in CD patients, supporting patient acceptance and feasibility of precision smoking cessation in this population. To reduce smoking in CD, therapies such as MIC are needed to maximize efficacy and minimize side effects.

## Introduction

Inflammatory bowel disease (IBD), including Crohn’s disease (CD) and ulcerative colitis (UC) are chronic relapsing and remitting inflammatory disorders of the bowel that affect 1.6 million people in the US alone [1]. Tobacco use is associated with an increased risk of developing CD, and is a strong risk factor for disease severity in established CD [2, 3]. Individuals with CD who smoke have more frequent relapses [4–6], suffer more complications [7], and require surgery more often than those who do not smoke [8, 9]. CD smokers require more immunosuppressive therapy to control their disease [10], and have higher disease related costs than non-smokers [11].

Several studies have shown that smoking cessation improves disease course in CD [12–14], but little has been studied about smoking cessation methods, particularly, the feasibility of a metabolism-informed approach in patients with CD. Both U.S. and European IBD guidelines recommend identifying smokers and actively promoting smoking cessation in CD patients [15–17]. Currently, however, there are no recommendations about how to specifically incorporate smoking cessation pharmacotherapy into CD care. Not surprisingly, rates of successful smoking cessation in individuals with CD remain low with published rates ranging from 12–37% [13, 18–20].

Effective, inexpensive, and safe cessation aids are readily available for smoking cessation in the general population. There are three FDA approved medications to assist with smoking cessation (nicotine replacement therapy, varenicline, and bupropion). Nicotine replacement therapy (NRT) replaces nicotine to reduce cravings in a variety of forms such as patches, gum, or lozenges [21]. NRT is affordable, has few side effects, and is available over the counter. Bupropion is a norepinephrine dopamine reuptake inhibitor used both as an antidepressant and smoking cessation aid [22]. Varenicline is a partial nicotinic acetylcholine receptor agonist [23]. All 3 agents have each been shown to improve quit rates compared to placebo [21–24], but varenicline in combination with behavioral therapy is the most effective method [25, 26].

To our knowledge, no studies have examined varenicline use specifically in a CD population. The lack of information on this medication is particularly relevant in the CD population because many common side effects of varenicline, including nausea, insomnia, and mood disturbances, are also frequently seen in CD [26]. Although generally well tolerated, up to 30% of those who take varenicline experience nausea, 12% experience abnormal dreams, 10% experience insomnia, and approximately 4% report a neuropsychiatric side effect such as agitation or mood disturbance [26].

Smoking cessation is a rapidly progressing field with recent advancements in precision approaches to cessation therapy based on metabolism informed care (MIC). Recent smoking literature notes that an individual’s rate of nicotine metabolism predicts nicotine dependence, cessation success, and outcomes with pharmacotherapy [27–32]. Nicotine is metabolized by the cytochrome P450 enzyme CYP2A6 to cotinine and subsequently 3-hydroxycotine. The nicotine metabolite ratio (NMR) is the ratio of nicotine breakdown products 3-hydroxycotinine to cotinine and is a biomarker of the rate of nicotine metabolism [33]. In a pivotal randomized controlled trial, smokers who are “normal” metabolizers of nicotine (NMR value ≥ 0.31) demonstrated a two-fold improvement in cessation rates when treated with varenicline. In this study, 4.9 normal metabolizers needed to be treated with varenicline for one cessation event whereas 26 had to be treated with NRT for each cessation event [34]. By contrast, cessation rates were equal (number needed to treat of 8.1 for varenicline and 10.3 for NRT patch for each cessation) with varenicline and NRT in “slow” metabolizers (NMR value < 0.31). Slow metabolizers also reported more side effects with varenicline compared to placebo [34]. These findings provide a rationale for MIC, an approach that matches normal metabolizers with non-nicotine-based therapy (preferably varenicline) and slow metabolizers with NRT patch. Due to the increased risk of disease complications with continued smoking, the CD population is ideal for a metabolism-based approach for smoking cessation.

Precision methods such as MIC are the future of smoking cessation and it is important to evaluate whether distinct, high risk populations such as CD patients are accepting of these therapies. For this reason, we sought to determine the feasibility, acceptance, and tolerance of metabolism informed smoking cessation therapy specifically within the CD population. Our first aim was to determine the current prevalence of smoking in a CD population through an observational cohort. Our second aim was to then assess smokers’ attitudes toward a personalized metabolism-informed approach to smoking cessation in an IBD center by quantifying the extent to which MIC improves the match rates between NMR and pharmacotherapy as compared to usual care in CD patients enrolled into a pilot randomized controlled trial. As secondary endpoints, we evaluated self-reported rates of medication use, decrease in cigarette consumption, and self-reported side effects associated with smoking cessation medications in each group.

## Methods

### Initial survey

Before beginning a precision smoking cessation intervention, we first sought to determine the ongoing prevalence of smoking in our IBD population. We analyzed the self-reported smoking status of 1098 consecutive individuals seen in the IBD center at Vanderbilt University Medical Center from October 2015 until January 2016. The IBD center is a tertiary care subspecialty clinic which sees approximately 6,500 patients with Crohn’s disease and ulcerative colitis. As part of usual care, individuals were asked to self-report if they were currently smoking, were a former smoker, or had never smoked. We recorded the diagnosis for each patient as assigned by the treating physician. For each patient with CD, we obtained clinical disease activity by the Harvey Bradshaw index (HBI) [35], IBD specific health related quality of life by the Short IBD Questionnaire (SIBDQ) [36], and depression screening with the Patient Health Questionnaire (PHQ-9) [37].

### Trial design

Based on the high prevalence of smoking observed in our initial survey, starting in June 2016 daily smokers with CD (n = 32) were recruited from the IBD center as part of a larger pilot study of MIC in chronic disease [38]. Written informed consent was obtained. The parent study was registered with ClinicalTrials.gov (identifier NCT03227679). Registration was initially delayed but the trial was registered as soon as the study team became aware of the delay. The authors confirm that all ongoing and related trials are registered. Here we analyze only the subset of patients with CD to investigate the feasibility of a personalized smoking cessation program specifically in a CD population.

### Ethical considerations

The study protocols were approved by the Vanderbilt University Medical Center Institutional Review Board (IRB # 151110 for smoking prevalence study approved on 8/17/2015 and IRB #160560 for MIC intervention approved on 5/4/2016). All methods were performed in accordance with relevant guidelines and regulations for human subject research. Data collection for IRB #151110 ran from 10/1/2015 to 1/21/2016. Enrollment for IRB #160560 began on 6/13/2016 and was completed 9/27/2016. The last follow up was competed 4/3/2017.

### Eligibility for smoking cessation intervention

IBD center patients who reported active smoking as part of their clinic intake screening were approached in person by study staff for eligibility screening and consent. Eligible subjects included adult (>18 years of age) daily smokers (≥5 cigarettes per day) seen in the IBD clinic who had no contraindications to taking at least two out of three smoking cessation medications (bupropion, varenicline, or NRT). Contraindications to each medication are listed in **S1 Table**. Exclusion criteria included the inability to give informed consent; serious psychiatric condition as defined by hospitalization for a psychiatric condition in the past year; unstable substance use disorder (e.g., alcohol dependence, opioid dependence) requiring hospitalization in the past year; seizure disorder; life expectancy <12 months; pregnant or breastfeeding; no access to a telephone or inability to communicate by telephone; or unable to speak and read English. Stable depression was not excluded. Because levels of nicotine metabolites fall below detection over time, patients who had been completely abstinent from cigarettes for >3 days were excluded.

### Intervention and medication provisions

All participants had blood drawn for nicotine metabolite measurement (ARUP laboratories; Salt Lake City, Utah). Serum nicotine metabolite testing is inexpensive and widely available. All participants received a telephone-based cessation intervention 1–2 weeks after enrollment from an IBD physician (D.B.B or E.A.S). Participants in both treatment groups received smoking cessation in the form of motivational interviewing, information about all 3 FDA approved smoking cessation aids, and referral to a free telephone cessation line (Quitline) from the IBD physician. Tobacco Quitlines are telephone or internet-based counseling services that are available free of charge in every state in the U.S. as well as many European countries [39, 40]. In the usual care group, participants were educated regarding smoking cessation medications, including efficacy and common side effects, and co-selected a medication with the IBD physician from those they were medically eligible to receive. In the usual care group both the IBD physician and participant were blinded to the NMR status. Procedures for the MIC arm were identical to usual care except the IBD physician was un-blinded to the NMR value and recommended NMR-informed choice of pharmacotherapy. Slow nicotine metabolizers (NMR < 0.31) were recommended NRT. Normal nicotine metabolizers (NMR ≥ 0.31) were recommended a non-nicotine based method (varenicline as first line or bupropion if not medically eligible for varenicline) [34]. MIC participants were made aware of the NMR results and the metabolism-based recommendation, but could choose any medication for which they were medically eligible. All participants in both groups were provided a 90-day supply of whichever medication (varenicline, bupropion, or NRT) was chosen.

### Outcomes and follow up

Upon enrollment, patients were given an initial survey detailing smoking history, smoking related traits (e.g. smoking intensity, measures of addiction, use of other tobacco products). Follow-up assessments were conducted at 1-month, 3-months, and 6-months after enrollment. Follow-up assessments included self-reported confidence to quit smoking, medication use, 7-day point-prevalence abstinence and/or cigarette consumption, satisfaction with care received in the study, and a symptom/side effect survey.

### Randomization

As part of the parent study, patients were randomly assigned in a 1:1 ratio to either usual care or MIC by stratified permuted block in blocks of 10. Randomization was stratified by cigarettes per day (greater than or less than or equal to 10 cigarettes per day). The randomization was assigned by computer generation at enrollment within our secure REDCap database. Randomization was not concealed to participants or researchers as NMR lab values were used for medication selection only in the MIC group. One patient was not randomized due to the NMR lab not being available due to lab error. This participant completed initial surveys but was excluded from further participation and analysis. One additional patient was randomized but could not be contacted to discuss medication options. Due to the small size of our pilot study, we report per protocol analysis of this randomized individual.

### Statistical analysis

Categorical variables were reported using percentages and compared using the Chi-squared with Fisher’s exact test applied when appropriate. Continuous variables were reported using mean and standard deviation and compared using the Mann-Whitney U test for two comparisons or the Kruskal-Wallis test for 3 or more comparisons. Paired analysis between baseline and 6 months was performed using the Wilcoxon Signed-rank test. All analysis was performed using STATA version 14.2 (StataCorp, College Station, TX).

## Results

### Prevalence and characteristics of self-reported smokers with Crohn’s disease

**Fig 1:**
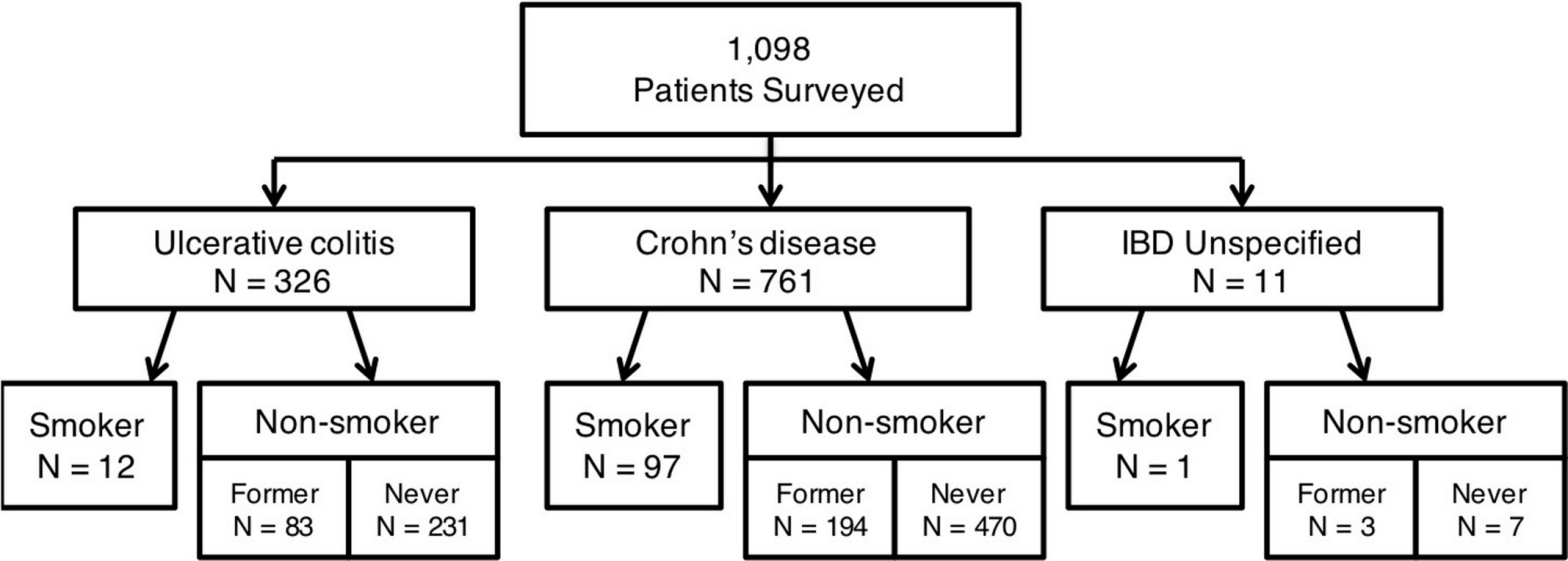
Prevalence of smoking in inflammatory bowel disease. The prevalence of cigarette smoking was determined over 1098 consecutive inflammatory bowel disease (IBD) specialty clinic visits over a 3-month period. IBD diagnosis was determined based on the diagnosis of the treating IBD physician. Smoking status was determined by self-report.

Prior to the initiation of our randomized intervention we desired to define the prevalence of smoking in our IBD population. Of 1098 consecutive patients surveyed, 761 (69.3%) had CD, 326 (29.7%) had UC, and 11 (1.0%) had indeterminate colitis. A total of 110 (10%) patients were active smokers with 97 (88.2% of all smokers) having a diagnosis of CD. The distribution of active smokers by diagnosis is shown in **Fig 1**.

The characteristics of CD smokers compared with non-smokers are shown in **Table 1.** Current and former smokers were older than never smokers. Clinical CD activity, by the HBI, and depressive symptoms, by the PHQ-9 score, were significantly higher in active smokers. Health related quality of life, by SIBDQ score, was significantly decreased in active smokers.

**Table 1:** Baseline demographics and disease indicators in current, former, and never smokers with Crohn’s disease.

	Current Smokers (n = 97)	Former Smokers (n = 194)	Never Smokers (n = 479)	p-value
Male Sex, n (%)	42 (43.3)	84 (43.3)	189 (39.5)	0.785
Age, mean (SD)	43.2 (12.6)	48.1 (15.5)	37.1 (14.0)	<0.001
HBI, mean (SD)	5.8 (5.6)	4.9 (6.0)	3.5 (4.2)	<0.001
SIBDQ, mean (SD)	46.5 (13.1)	53.4 (13.4)	55.0 (12.6)	<0.001
PHQ-9, mean (SD)	7.9 (5.8)	6.0 (7.4)	4.8 (6.1)	0.001
Biologic Use, n (%)	75 (77.3)	148 (76.3)	372 (77.7)	0.702

HBI = Harvey Bradshaw Index, PHQ-9 = Patient Health Questionnaire, SIBDQ = Short IBD Questionnaire. PHQ-9 scores were significantly increased while SIBDQ scores were significantly decreased across smoking status. The Kruskal-Wallis test was used for continuous variables and Chi-Squared test for categorical variables.

### NMR intervention study population

**Fig 2:**
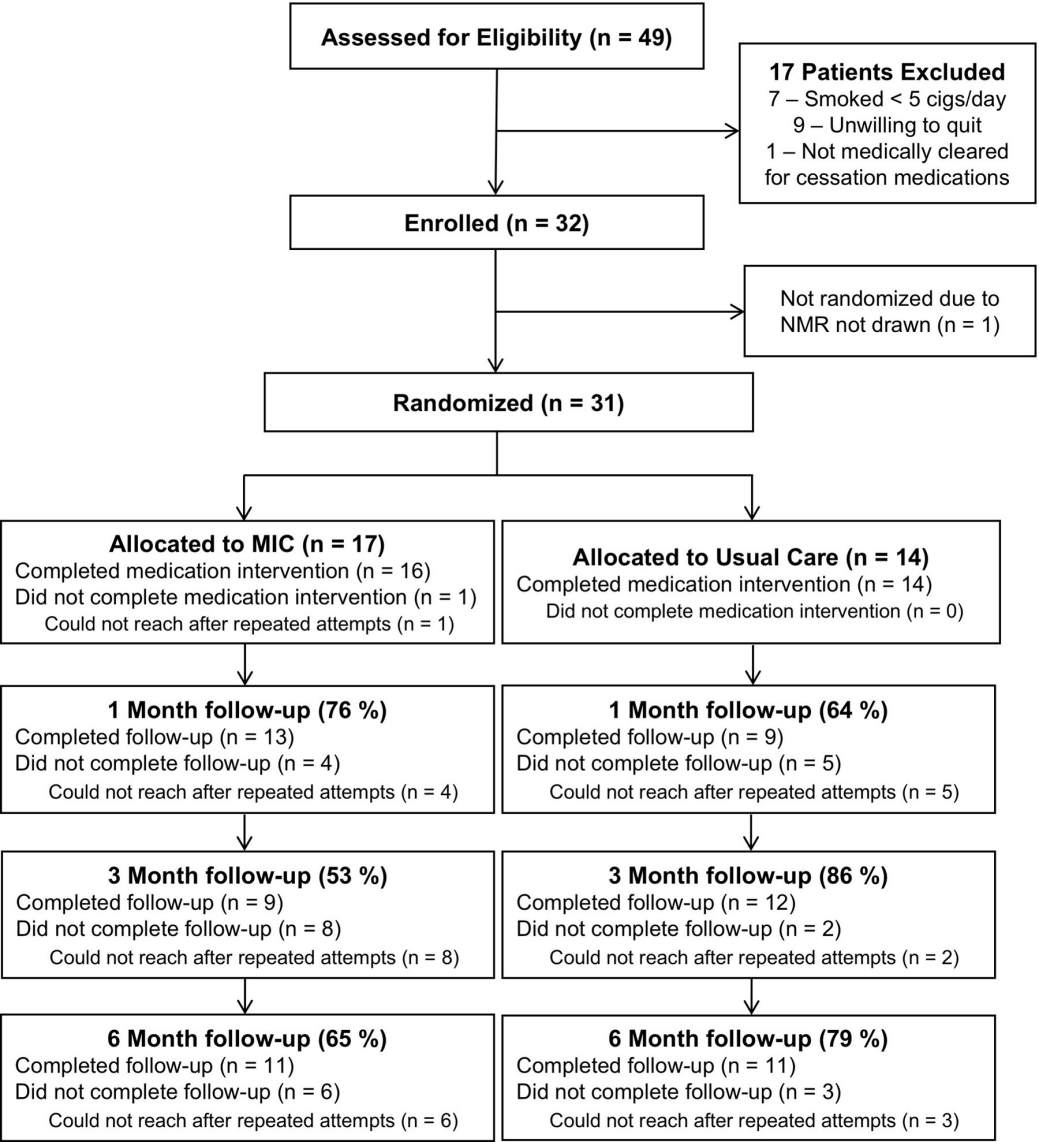
Flow of participants in the nicotine metabolite ratio intervention. Thirty-two subjects participated in this randomized pilot intervention using nicotine metabolite ratio (NMR) to guide smoking cessation.

After observing a high ongoing prevalence of smoking in CD despite usual care, we recruited a total of 32 smokers with CD to participate in a pilot study of MIC guided by NMR. The flow of participants in this study is shown in **Fig 2**.

Overall, 22 (71.0%) enrolled patients completed the 1-month follow up, 21 (67.7%) completed 3-month follow up, and 22 (71.0%) completed 6-month follow up. Five (16.1%) patients did not respond to telephone surveys, but on review of medical records, none self-reported smoking cessation to their providers during the study follow up. **Table 2** shows baseline characteristics of CD patients who participated in the randomized intervention. Nicotine metabolites were detected in all subjects (**S1 Fig**).

**Table 2:** Baseline characteristics of smoking cessation intervention participants.

	All (n = 32)	MIC (n = 17)	Usual Care (n = 14)
Age, mean (SD)	44 (11.1)	43 (10.0)	45 (12.9)
Male Sex, n (%)	15 (46.9)	7 (41.2)	8 (57.1)
Harvey Bradshaw Index, mean (SD)	5.71 (5.0)	4.3 (4.4)	7 (5.3)
SIBDQ, mean (SD)	45.37 (13.8)	47.00 (38.7)	44.08 (11.8)
PHQ-9, mean (SD)	9.81 (10.6)	9.88 (13.4)	9.14 (6.7)
Normal Metabolizer by NMR, n (%)	18 (56.3)	8 (47.1)	10 (71.4)
Cigarettes per day, mean (SD)	18.5 (9.2)	17.5 (10.6)	20.3 (7.3)
Income			
<$25,000	9 (28.1)	2 (11.7)	6 (42.9)
$25,000–50,000	12 (37.5)	8 (57.1)	4 (28.6)
$50,000–75,000	4 (12.5)	4 (23.5)	0 (0)
>$75,000	6 (18.8)	2 (11.8)	4 (28.6)
Don’t know/Decline	1 (3.1)	1 (5.9)	0 (0)
Education			
High School or Less	13 (40.6)	6 (35.3)	7 (50.0)
Vocational	5 (15.6)	2 (11.8)	3 (21.4)
College or College graduate	14 (43.8)	9 (52.9)	4 (28.6)
IBD Medications, n (%)			
5-ASA	3 (9.4)	1 (5.9)	2 (14.3)
Immunomodulator	10 (31.2)	4 (23.5)	6 (42.9)
Anti-TNF-α	22 (68.8)	13 (76.5)	8 (57.1)
Vedolizumab	5 (15.6)	3 (17.6)	2 (14.3)
Ustekinumab	1 (3.1)	0 (0)	1 (7.1)
Prednisone	4 (12.5)	2 (11.8)	2 (14.3)

SIBDQ = Short IBD Questionnaire, PHQ-9 = Patient Health Questionnaire-9. One patient in the MIC group did not have an NMR value and was not prescribed a medication due to lab error.

### Baseline attitudes on smoking cessation

Twenty-four participants (75.0%) agreed or strongly agreed that they know what treatments are available to help them quit smoking, despite only 5 (15.6%) having used a cessation medication and 3 (9.4%) a behavioral aid within 6 months. The distribution of these 24 patients was similar in both treatment groups (11 MIC, 12 usual care, 1 not randomized). Despite having CD, only 5 (15.6%) agreed or strongly agreed that they were at higher risk of smoking related illness than other smokers. The distribution was similar between treatment groups (2 MIC, 2 usual care, and 1 not randomized). Medication was accepted by 30 (93.7%) and Quitline referral by 28 (87.5%) patients. Of the two patients who did not accept medication one could not be randomized due to lab error and the other in the MIC group could not be reached by telephone. Three patients who did not accept Quitline services were all in the MIC group and 1 was not randomized. Five patients (4 usual care, 1 MIC) reported that their medication choice (all avoided varenicline) was based on a concern for potential side effects. Detailed survey information based on randomization groups is available in (**S2 Table**).

### Medication matching and satisfaction with metabolism informed care

Overall, 94% (16/17) of MIC participants randomized and 100% (16/16) who completed the medication phone call accepted a prescription that matched their NMR status (**Table 3**). By comparison, 9 of 14 usual care participants (64.3%) happened to receive (i.e. by chance) smoking medication that matched their NMR status (p = 0.01).

**Table 3:** Matching of NMR pharmacotherapy by treatment arm.

		MIC (n = 16)		Usual Care (n = 14)	
		Normal (n = 8)	Slow (n = 8)	Normal (n = 10)	Slow (n = 4)
Medication prescribed	Varenicline	7 (87.5%)	0 (0.0%)	6 (60.0%)	3 (75.0%)
	Bupropion	1 (12.5%)	0 (0.0%)	2 (20.0%)	0 (0.0%)
	Nicotine Patch	0 (0.0%)	8 (100%)	2 (20.0%)	1 (25.0%
NMR Medication Matching*		16/16 (100%)		9/14 (64.3%)	

One participant in the MIC group could not be reached by telephone to prescribe a medication and was excluded from per protocol analysis. p-values are calculated by the Fisher’s exact test.

*(p = 0.01)

Overall, participants had positive baseline perceptions of MIC (**Table 4**). The majority of patients reported satisfaction with the intervention and felt like they had received the best possible treatment to help them quit smoking at all 3 time points (**Table 5**). The distribution of satisfaction was similar in both the usual care and MIC groups (**Table 5**).

**Table 4:** Participant baseline perceptions about metabolism informed care.

Baseline Perceptions, n (%)				
Survey Question	Response	All (n = 32)	MIC (n = 17)	Usual care (n = 14)
I approve of using tests to determine how my body breaks down nicotine to help me quit smoking.	Strongly agree/Agree	31 (96.9%)	16 (94.1%)	14 (100%)
	Neither agree nor disagree/ Disagree/ Strongly Disagree	1 (3.1%)	1 (5.9%)	0 (0%)
	Don't know/ Decline	0 (0%)	0 (0%)	0 (0%)
The development of blood tests to help match patients with the drugs that might work best for them is a positive medical progress.	Strongly agree/Agree	28 (87.5%)	14 (82.4%)	13 (92.9%)
	Neither agree nor disagree/ Disagree/ Strongly Disagree	2 (6.3%)	1 (5.9%)	1 (7.1%)
	Don't know/ Decline	2 (6.3%)	2 (11.8%)	0 (0%)
If a blood test told me that I might have a more difficult time quitting smoking than some other people I wouldn't even bother trying to quit.	Strongly agree/Agree	1 (3.1%)	1 (5.9%)	0 (0%)
	Neither agree nor disagree/ Disagree/ Strongly Disagree	28 (87.5%)	14 (82.4%)	13 (92.9%)
	Don't know/ Decline	3 (9.4%)	2 (11.8%)	1 (7.1%)
The idea of knowing how my body breaks down nicotine frightens me.	Strongly agree/Agree	3 (9.4%)	2 (11.8%)	1 (7.1%)
	Neither agree nor disagree/ Disagree/ Strongly Disagree	27 (84.4%)	13 (76.5%)	13 (92.9%)
	Don't know/ Decline	2 (6.3%)	2 (11.8%)	0 (0%)

Participants completed questionnaires about their baseline perceptions of MIC at baseline. One patient (MIC) was lost to follow up before medication call.

**Table 5:** Participant reported satisfaction with intervention at 1-, 3-, and 3-month follow ups.

Satisfaction on Follow Up, n (%)				
Survey Question	Response	All	MIC	Usual care
**1 Month Follow Up**		**n = 22**	**n = 13**	**n = 9**
I am satisfied with the smoking treatment I have received from the study.	Strongly agree/Agree	20 (90.9%)	10 (76.9%)	9 (100%)
	Neither agree nor disagree/ Disagree/ Strongly Disagree	3 (13.6%)	3 (23%)	0 (0%)
	Don't know/ Decline	0 (0%)	0 (0%)	0 (0%)
The smoking treatment I received from the study was tailored specifically to me.	Strongly agree/Agree	19 (86.4%)	12 (92.3%)	7 (77.8%)
	Neither agree nor disagree/ Disagree/ Strongly Disagree	2 (9.1%)	1 (7.7%)	1 (11.1%)
	Don't know/ Decline	1 (4.5%)	0 (0%)	1 (11.1%)
I feel like I have received the best treatment possible to help me quit smoking.	Strongly agree/Agree	22 (100%)	13 (100%)	9 (100%)
	Neither agree nor disagree/ Disagree/ Strongly Disagree	0 (0%)	0 (0%)	0 (0%)
	Don't know/ Decline	0 (0%)	0 (0%)	0 (0%)
**3 Month Follow Up**		**n = 21**	**n = 9**	**n = 12**
I am satisfied with the smoking treatment I have received from the study.	Strongly agree/Agree	19 (90.5%)	8 (88.9%)	11 (91.7%)
	Neither agree nor disagree/ Disagree/ Strongly Disagree	2 (9.5%)	1 (11.1%)	1 (8.3%)
	Don't know/ Decline	0 (0%)	0 (0%)	0 (0%)
The smoking treatment I received from the study was tailored specifically to me.	Strongly agree/Agree	19 (90.5%)	8 (88.9%)	11 (91.7%)
	Neither agree nor disagree/ Disagree/ Strongly Disagree	1 (4.8%)	0 (0%)	1 (8.3%)
	Don't know/ Decline	1 (4.8%)	1 (11.1%)	0 (0%)
I feel like I have received the best treatment possible to help me quit smoking.	Strongly agree/Agree	17 (81.0%)	7 (77.8%)	10 (83.3%)
	Neither agree nor disagree/ Disagree/ Strongly Disagree	2 (9.5%)	1 (11.1%)	1 (8.3%)
	Don't know/ Decline	2 (9.5%)	1 (11.1%)	1 (8.3%)
**6 Month Follow Up**		**n = 22**	**n = 11**	**n = 11**
I am satisfied with the smoking treatment I have received from the study.	Strongly agree/Agree	18 (81.8%)	9 (81.8%)	9 (81.8%)
	Neither agree nor disagree/ Disagree/ Strongly Disagree	4 (18.2%)	2 (18.2%)	2 (18.2%)
	Don't know/ Decline	0 (0%)	0 (0%)	0 (0%)
The smoking treatment I received from the study was tailored specifically to me.	Strongly agree/Agree	20 (90.9%)	9 (81.8%)	11 (100%)
	Neither agree nor disagree/ Disagree/ Strongly Disagree	2 (9.1%)	2 (18.2%)	0 (0%)
	Don't know/ Decline	0 (0%)	0 (0%)	0 (0%)
I feel like I have received the best treatment possible to help me quit smoking.	Strongly agree/Agree	22 (100%)	11 (100%)	11 (100%)
	Neither agree nor disagree/ Disagree/ Strongly Disagree	0 (0%)	0 (0%)	0 (0%)
	Don't know/ Decline	0 (0%)	0 (0%)	0 (0%)

Participants completed questionnaires about satisfaction with the intervention at 1-, 3-, and 6-months. For each time point, n represents the number of participants who responded to questionnaires.

### Self-reported medication side effects and compliance with interventions

**Fig 3:**
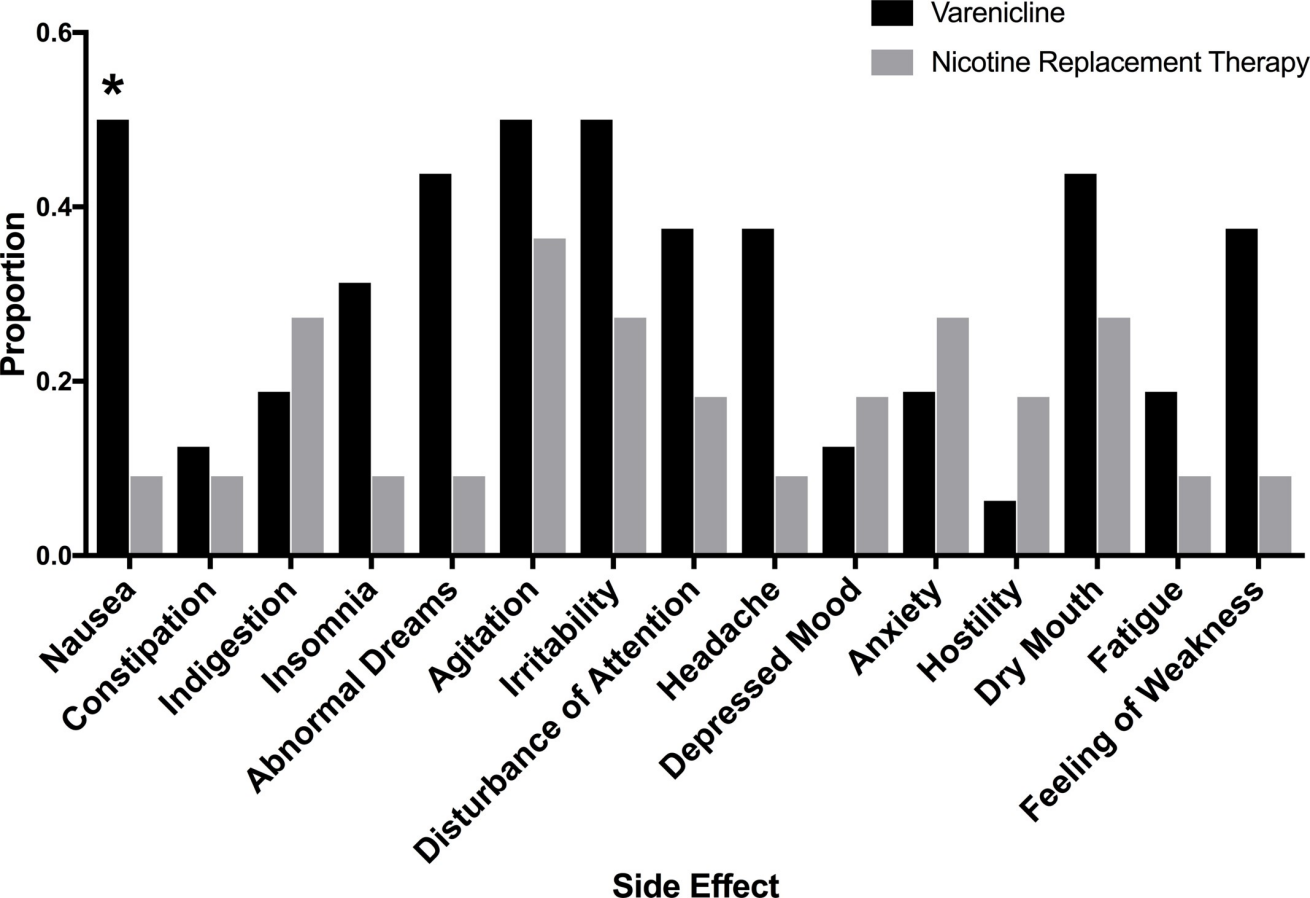
Side effects reported based on medication prescribed. Side effects are reported as the proportion of patients reporting new or worsening symptoms from baseline while on varenicline (black) compared with nicotine replacement therapy (grey). * p = 0.04 by the Fisher’s exact test. No other comparison between varenicline and NRT was statistically significant. Due to the low number of individuals on bupropion (n = 3) these were excluded from the figure. Participants on bupropion reported mild headache, depressed mood, and hostility (all n = 1).

In general, side effects were reported more commonly in the varenicline group. Patients on varenicline reported significantly higher rates of nausea (p = 0.04) than those on NRT (**Fig 3**). Sample size did not permit stratification of individual side effects by NMR status. Participants on bupropion reported mild headache, depressed mood, and hostility (all n = 1).

Six of 16 patients (37.5%) prescribed varenicline discontinued due to side effects, whereas, no participants prescribed NRT or bupropion discontinued medication due to side effects. The rate of discontinuation due to side effects, however, was similar in MIC compared to usual care arms (n = 2 vs. n = 4). Sleep-related (insomnia, abnormal dreams; n = 3), neuropsychiatric (irritability, distorted thoughts, agitation; n = 3), and GI related side effects (nausea; n = 3) were reported as reasons for discontinuation of varenicline.

In our CD population, 3 of 3 (100%) of slow metabolizers on varenicline reported a side effect, and 2 of 3 (66.7%) slow metabolizers discontinued the medication. While our sample of slow metabolizers on varenicline is small these rates are significantly higher than expected. Comparatively, 11 of 13 (84.6%) of normal metabolizers on varenicline reported a side effect, and 4 of 13 (30.8%) discontinued the medication.

In addition to those who discontinued due to side effects, 2 participants taking varenicline self-discontinued due to perceived ineffectiveness and 1 participant prescribed NRT chose not to start medication due to life stressors. Based on direct feedback from Quitlines, only 2 participants formally enrolled in telephone or internet-based counseling.

### Smoking outcomes

**Fig 4:**
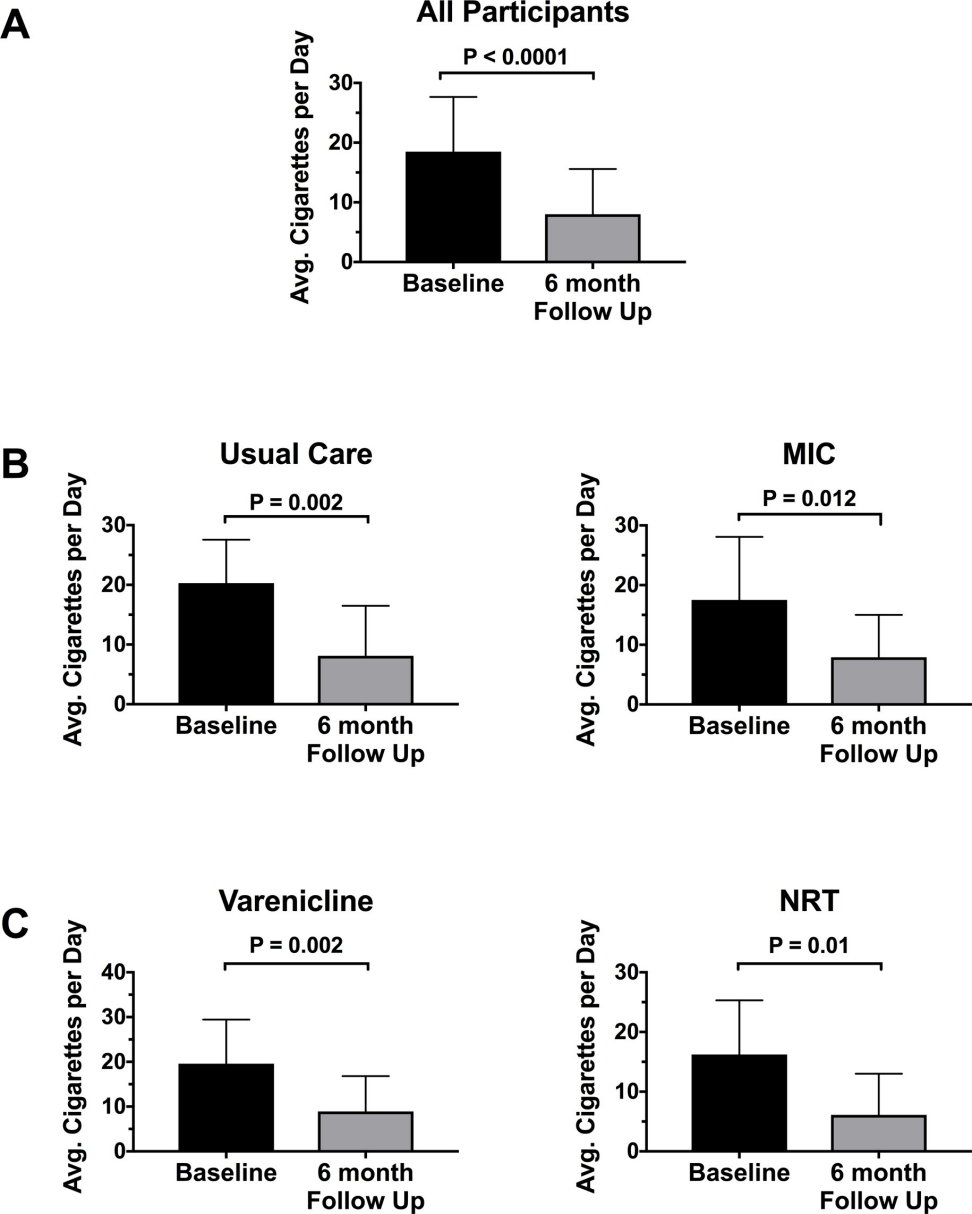
Cigarettes per day at baseline and 6-month follow up based on (A) all participants (B) treatment arm and (C) medication choice. Average cigarettes per day for each participant was determined from patient recall of the previous 7 days. P values are determined by a Wilcoxon Signed-Rank test. Error bars represent standard deviation. Only one of three participants on bupropion completed 6-month follow up.

Five (16.1%) patients reported complete smoking cessation at 6 months. Of these, 3 reported cessation at all three follow up time points. Two reported cessation at both the 3- and 6-month follow up time points. No participant who reported cessation at earlier time points reported restarting smoking. A total of 15 patients (48.4%) reached a combined endpoint of ≥50% decrease in cigarettes smoked per day or complete cessation at 6 months. An additional 3 patients reported a ≥50% decrease in cigarettes smoked per day at the 1- or 3-month follow-ups but were lost to additional follow up. The combined endpoint at 6 months did not differ significantly in MIC vs. usual care groups (n = 6 vs. n = 9). Decreasing cigarette consumption ≥50% at 6 months was more common with varenicline (n = 10) compared with those who took NRT (n = 5) or bupropion (n = 0). Overall, participants reported smoking fewer cigarettes at 6 months across both treatment arms as well as in the subgroups who received varenicline and NRT (**Fig 4)**.

## Discussion

Despite a well-known need for smoking cessation in the CD population, we found a high prevalence of ongoing active smoking in our tertiary IBD center, emphasizing the need for improved cessation methods in this population. We also report low acknowledgement of the risks of smoking in CD as well as low rates of pre-intervention cessation aid utilization in our pilot study population. This is the first study to examine MIC, a precision treatment for smoking cessation, in patients with CD. MIC for smoking cessation is highly endorsed by CD patients, who demonstrated a high degree of willingness to accept NMR-guided pharmacotherapy. In addition, we found higher-than-expected proportions of CD patients had side effects to varenicline resulting in medication discontinuation.

Despite the preservation of patient autonomy to choose any FDA approved medication for which they were medically eligible, participants in the MIC group consistently chose the medication that matched their NMR status. Because varenicline is generally considered more effective than NRT (when NMR is not considered) we would anticipate its use to increase. However, there are significant differences in the cost and side effect profiles of these medications and this seems to be particularly pronounced in the CD population. While no patient in our study experienced a severe or life-threatening adverse event, the rate of side effects resulting in discontinuation of varenicline was much higher than expected. In the recent EAGLES study of over 8000 patients receiving varenicline, NRT, or bupropion, only 5.8% of patients without an underlying psychiatric disorder and 10.6% of patients with an underlying psychiatric disorder discontinued varenicline as a result of a side effect [26]. In the current study, the rate of 38% of CD participants who discontinued varenicline due to side effects is 3 to 6-fold than prior reports.

It is possible that the baseline concerns with sleep, nausea, and neuropsychiatric symptoms, which are common in CD, may result in a lessened tolerance of these side effects of varenicline. It is notable that none of the 6 patients who prematurely discontinued varenicline due to side effects quit smoking, suggesting that the high rate of side effects shown in our study may have decreased rates of cessation. CD may be an ideal population for MIC smoking cessation as it could allow providers to reserve varenicline for those who are most likely to benefit, while sparing slow metabolizers the potential for side effects and cost. Approximately 44% of our population consists of slow metabolizers (using the 0.31 NMR threshold); thus, MIC could help avoid varenicline in a substantial number of CD patients without affecting cessation efficacy.

These results further emphasize the potential difficulty of cessation for CD smokers who are normal nicotine metabolizers. Normal metabolizers tend not to be able to quit smoking with nicotine patch [34] and those with CD also appear to have higher than expected rates of varenicline discontinuation. Determining a therapeutic alternative for those unable to tolerate varenicline is not straightforward. Bupropion was offered in this study design as a non-nicotine alternative, although there is limited evidence for superior efficacy of bupropion in normal metabolizers. Extending the duration of NRT did not improve quit rates in a general population of smokers [41]. Increasing the dose of nicotine patch in normal metabolizers modestly improved quit rates relative to usual dose in the general smoking population, but this has not yet been tested in an adequately powered randomized trial [42]. As our NRT-exposed patients also reported relatively high rates of side effects, these strategies also warrant testing specifically in a CD population.

As an initial feasibility study of MIC in a specialty population our sample size is limited (n = 32) to the subset of CD patients included in the parent study. As a sub-analysis, this study was aimed at demonstrating feasibility by measuring satisfaction and acceptance of the MIC smoking cessation intervention in an IBD setting. As such, it is not powered to show definitive differences in this subset. In addition, the single tertiary IBD center setting may not be fully applicable to other clinics, although this site does reflect a broad, multi-state catchment area. Similarly, we enrolled CD patients who are motivated to quit smoking which may not be representative of the smoking CD population as a whole, but is likely representative of those patients who would participate in future interventions. While our overall completion rates for follow up surveys was 70%, loss of follow up in this small study may have further decreased power of our secondary outcomes. This study is a sub-analysis of a larger randomized controlled trial and thus the sub-set of patients we have analyzed may be subject to bias of unequal randomization in this small subset. Our study design required unblinding participants in the MIC group to the results of their NMR results. While this may have influenced their confidence to quit smoking it presents a pragmatic approach to the use of NMR testing in a real-world environment. Importantly, this pilot RCT did not include a placebo control. Thus the role of the process of smoking cessation itself, which is known to cause numerous unpleasant experiences, is not evaluated here [26].

In conclusion, this is the first study to our knowledge to demonstrate the feasibility of MIC, a precision approach to smoking cessation, for a subspecialty chronic disease population. Despite following evidence-based guidelines to counsel on smoking cessation, rates of smoking in CD remain high and the baseline use of cessation aids remain low in our tertiary IBD center. Based on our data, more than half of CD smokers are normal nicotine metabolizers and should receive varenicline as a first line medication in order to maximize their likelihood of cessation. However, the suggestion that CD patients may be less tolerant of varenicline than the general population could potentially complicate precision cessation efforts in CD. Further investigation of precision approaches to smoking cessation is needed to determine their impact on CD populations.

## Supporting information

S1 Table Contraindications to smoking pharmacotherapy used for study eligibility. (DOCX)

S2 Table Study raw data. De-identified raw data for all 32 participants is provided. Dates, demographics, and any potentially identifying information has been removed.
(XLS)

S1 Fig Distribution of NMR values for the entire cohort of participants. NMR was calculated as the ratio of 3-hydroxycotinine to cotinine as measured in serum.
(DOCX)

S1 Protocol (DOCX)

S1 Checklist CONSORT 2010 checklist of information to include when reporting a randomised trial*. (DOC)

## References

[1] Shivashankar R, Tremaine WJ, Harmsen WS, Loftus EV Jr. Incidence and Prevalence of Crohn's Disease and Ulcerative Colitis in Olmsted County, Minnesota From 1970 Through 2010. Clin Gastroenterol Hepatol. 2017;15(6):857–63. 10.1016/j.cgh.2016.10.039 27856364 PMC5429988

[2] Mahid SS, Minor KS, Soto RE, Hornung CA, Galandiuk S, editors. Smoking and inflammatory bowel disease: a meta-analysis. Mayo Clin Proc. 2006;82(7):890. 10.4065/81.11.1462 17120402

[3] Sutherland LR, Ramcharan S, Bryant H, Fick G. Effect of cigarette smoking on recurrence of Crohn's disease. Gastroenterology. 1990;98(5 Pt 1):1123–8. 2323505 10.1016/0016-5085(90)90324-t

[4] Duffy LC, Zielezny MA, Marshall JR, Weiser MM, Byers TE, Phillips JF, et al Cigarette smoking and risk of clinical relapse in patients with Crohn's disease. Am J Prev Med. 1990;6(3):161–6. 2397140

[5] Russel MG, Nieman FH, Bergers JM, Stockbrugger RW. Cigarette smoking and quality of life in patients with inflammatory bowel disease. South Limburg IBD Study Group. European journal of gastroenterology & hepatology. 1996;8(11):1075–81. 8944369 10.1097/00042737-199611000-00009

[6] Breuer-Katschinski BD, Hollander N, Goebell H. Effect of cigarette smoking on the course of Crohn's disease. Eur J Gastroenterol Hepatol. 1996;8(11):1075–81. 10.1097/00042737-199611000-00009 8724021

[7] Lindberg E, Järnerot G, Huitfeldt B. Smoking in Crohn's disease: effect on localisation and clinical course. Gut. 1992;33(6):779–82. 10.1136/gut.33.6.779 1624159 PMC1379335

[8] Cottone M, Rosselli M, Orlando A, Oliva L, Puleo A, Cappello M, et al Smoking habits and recurrence in Crohn's disease. Gastroenterology. 1994;106:643 10.1016/0016-5085(94)90697-1 8119535

[9] Lunney PC, Kariyawasam VC, Wang RR, Middleton KL, Huang T, Selinger CP, et al Smoking prevalence and its influence on disease course and surgery in Crohn's disease and ulcerative colitis. Aliment Pharmacol Ther. 2015;42(1):61–70. 10.1111/apt.13239 25968332

[10] Cosnes J, Carbonnel F, Carrat F, Beaugerie L, Cattan S, Gendre J. Effects of current and former cigarette smoking on the clinical course of Crohn's disease. Aliment Pharmacol Ther. 1999;13(11):1403–12. 10.1046/j.1365-2036.1999.00630.x 10571595

[11] Severs M, Mangen MJ, van der Valk ME, Fidder HH, Dijkstra G, van der Have M, et al Smoking is Associated with Higher Disease-related Costs and Lower Health-related Quality of Life in Inflammatory Bowel Disease. J Crohns Colitis. 2017;11(3):342–52. 10.1093/ecco-jcc/jjw160 27647859

[12] Nunes T, Etchevers MJ, Garcia-Sanchez V, Ginard D, Marti E, Barreiro-de Acosta M, et al Impact of Smoking Cessation on the Clinical Course of Crohn's Disease Under Current Therapeutic Algorithms: A Multicenter Prospective Study. Am J Gastroenterol. 2016;111(3):411–9. 10.1038/ajg.2015.401 26856753

[13] Cosnes J, Beaugerie L, Carbonnel F, Gendre JP. Smoking cessation and the course of Crohn's disease: an intervention study. Gastroenterology. 2001;120(5):1093–9. 10.1053/gast.2001.23231 11266373

[14] To N, Gracie DJ, Ford AC. Systematic review with meta-analysis: the adverse effects of tobacco smoking on the natural history of Crohn's disease. Aliment Pharmacol Ther. 2016;43(5):549–61. 10.1111/apt.13511 26749371

[15] Ungar B, Levy I, Yavne Y, Yavzori M, Picard O, Fudim E, et al Optimizing Anti-TNF-alpha Therapy: Serum Levels of Infliximab and Adalimumab Are Associated With Mucosal Healing in Patients With Inflammatory Bowel Diseases. Clin Gastroenterol Hepatol. 2016;14(4):550-7.e2. 10.1016/j.cgh.2015.10.025 26538204

[16] Lichtenstein GR, Loftus EV, Isaacs KL, Regueiro MD, Gerson LB, Sands BE. ACG Clinical Guideline: Management of Crohn's Disease in Adults. Am J Gastroenterol. 2018;113(4):481–517. 10.1038/ajg.2018.27 29610508

[17] Gionchetti P, Dignass A, Danese S, Magro Dias FJ, Rogler G, Lakatos PL, et al 3rd European Evidence-based Consensus on the Diagnosis and Management of Crohn's Disease 2016: Part 2: Surgical Management and Special Situations. J Crohns Colitis. 2017;11(2):135–49. 10.1093/ecco-jcc/jjw169 27660342

[18] Leung Y, Kaplan GG, Rioux KP, Hubbard J, Kamhawi S, Stasiak L, et al Assessment of variables associated with smoking cessation in Crohn’s disease. Dig Dis Sci. 2012;57(4):1026–32. 10.1007/s10620-012-2038-2 22311366

[19] Nunes T, Etchevers MJ, Merino O, Gallego S, García-Sánchez V, Marín-Jiménez I, et al High smoking cessation rate in Crohn's disease patients after physician advice–The TABACROHN Study. J Crohns Colitis. 2013;7(3):202–7. 10.1016/j.crohns.2012.04.011 22626507

[20] Kennelly RP, Subramaniam T, Egan LJ, Joyce MR. Smoking and Crohn's disease: active modification of an independent risk factor (education alone is not enough). J Crohns Colitis. 2013;7(8):631–5. 10.1016/j.crohns.2012.08.019 23036508

[21] Silagy C, Lancaster T, Stead L, Mant D, Fowler G. Nicotine replacement therapy for smoking cessation. Cochrane Database Syst Rev. 2004;3:CD000146 10.1002/14651858.CD000146.pub2 15266423

[22] Hughes JR, Stead LF, Lancaster T. Antidepressants for smoking cessation. Cochrane Database Syst Rev. 2014;1:CD000031. 10.1002/14651858.CD000031.pub4 PMC7027688 24402784

[23] Gonzales D, Rennard SI, Nides M, Oncken C, Azoulay S, Billing CB, et al Varenicline, an α4β2 nicotinic acetylcholine receptor partial agonist, vs sustained-release bupropion and placebo for smoking cessation: a randomized controlled trial. JAMA. 2006;296(1):47–55. 10.1001/jama.296.1.47 16820546

[24] Jorenby D, Hays J, Rigotti N. A randomized controlled trial comparing the efficacy of varenicline, a novel α4β2 nicotinic acetylcholine receptor partial agonist, to bupropion and to placebo for smoking cessation. JAMA. 2006;296:56–63. 10.1001/jama.296.1.56 16820547

[25] Treating tobacco use and dependence: 2008 update U.S. Public Health Service Clinical Practice Guideline executive summary. Respir Care. 2008;53(9):1217–22. 18807274

[26] Anthenelli RM, Benowitz NL, West R, St Aubin L, McRae T, Lawrence D, et al Neuropsychiatric safety and efficacy of varenicline, bupropion, and nicotine patch in smokers with and without psychiatric disorders (EAGLES): a double-blind, randomised, placebo-controlled clinical trial. Lancet. 2016;387(10037):2507–20. 10.1016/S0140-6736(16)30272-0 27116918

[27] West O, Hajek P, McRobbie H. Systematic review of the relationship between the 3-hydroxycotinine/cotinine ratio and cigarette dependence. Psychopharmacology (Berl). 2011;218(2):313–22. 21597990 10.1007/s00213-011-2341-1

[28] Gambier N, Batt AM, Marie B, Pfister M, Siest G, Visvikis-Siest S. Association of CYP2A6*1B genetic variant with the amount of smoking in French adults from the Stanislas cohort. Pharmacogenomics J. 2005;5(4):271–5. 10.1038/sj.tpj.6500314 15940289

[29] Strasser AA, Benowitz NL, Pinto AG, Tang KZ, Hecht SS, Carmella SG, et al Nicotine metabolite ratio predicts smoking topography and carcinogen biomarker level. Cancer Epidemiol Biomarkers Prev. 2011;20(2):234–8. 10.1158/1055-9965.EPI-10-0674 21212060 PMC3077576

[30] Schnoll RA, Patterson F, Wileyto EP, Tyndale RF, Benowitz N, Lerman C. Nicotine metabolic rate predicts successful smoking cessation with transdermal nicotine: a validation study. Pharmacol Biochem Behav. 2009;92(1):6–11. 10.1016/j.pbb.2008.10.016 19000709 PMC2657225

[31] Ho MK, Mwenifumbo JC, Al Koudsi N, Okuyemi KS, Ahluwalia JS, Benowitz NL, et al Association of nicotine metabolite ratio and CYP2A6 genotype with smoking cessation treatment in African-American light smokers. Clin Pharmacol Ther. 2009;85(6):635–43. 10.1038/clpt.2009.19 19279561 PMC3698861

[32] Patterson F, Schnoll RA, Wileyto EP, Pinto A, Epstein LH, Shields PG, et al Toward personalized therapy for smoking cessation: a randomized placebo-controlled trial of bupropion. Clin Pharmacol Ther. 2008;84(3):320–5. 10.1038/clpt.2008.57 18388868

[33] Dempsey D, Tutka P, Jacob P, Allen F, Schoedel K, Tyndale RF, et al Nicotine metabolite ratio as an index of cytochrome P450 2A6 metabolic activity. Clin Pharmacol Ther. 2004;76(1):64–72. 10.1016/j.clpt.2004.02.011 15229465

[34] Lerman C, Schnoll RA, Hawk LW Jr., Cinciripini P, George TP, Wileyto EP, et al Use of the nicotine metabolite ratio as a genetically informed biomarker of response to nicotine patch or varenicline for smoking cessation: a randomised, double-blind placebo-controlled trial. Lancet Respir Med. 2015;3(2):131–8. 10.1016/S2213-2600(14)70294-2 25588294 PMC4480925

[35] Harvey RF, Bradshaw JM. A simple index of Crohn's-disease activity. Lancet. 1980;1(8167):514 10.1016/s0140-6736(80)92767-1 6102236

[36] Irvine EJ, Zhou Q, Thompson AK. The Short Inflammatory Bowel Disease Questionnaire: a quality of life instrument for community physicians managing inflammatory bowel disease. CCRPT Investigators. Canadian Crohn's Relapse Prevention Trial. Am J Gastroenterol. 1996;91(8):1571–8. 8759664

[37] Spitzer RL, Kroenke K, Williams JB. Validation and utility of a self-report version of PRIME-MD: the PHQ primary care study. Primary Care Evaluation of Mental Disorders. Patient Health Questionnaire. JAMA. 1999;282(18):1737–44. 10.1001/jama.282.18.1737 10568646

[38] Wells QS, Freiberg MS, Greevy RA Jr., Tyndale RF, Kundu S, Duncan MS, et al Nicotine Metabolism-informed Care for Smoking Cessation: A Pilot Precision RCT. Nicotine Tob Res. 2018;20(12):1489–96. 10.1093/ntr/ntx235 29059367 PMC6236077

[39] McAfee TA. Quitlines a tool for research and dissemination of evidence-based cessation practices. Am J Prev Med. 2007;33(6 Suppl):S357–67. 10.1016/j.amepre.2007.09.011 18021911

[40] Lichtenstein E, Zhu SH, Tedeschi GJ. Smoking cessation quitlines: an underrecognized intervention success story. Am Psychol. 2010;65(4):252–61. 10.1037/a0018598 20455619 PMC3169380

[41] Schnoll RA, Goelz PM, Veluz-Wilkins A, Blazekovic S, Powers L, Leone FT, et al Long-term nicotine replacement therapy: a randomized clinical trial. JAMA Intern Med. 2015;175(4):504–11. 10.1001/jamainternmed.2014.8313 25705872 PMC4410859

[42] Schnoll RA, Wileyto EP, Leone FT, Tyndale RF, Benowitz NL. High dose transdermal nicotine for fast metabolizers of nicotine: a proof of concept placebo-controlled trial. Nicotine Tob Res. 2013;15(2):348–54. 10.1093/ntr/nts129 22589423 PMC3545715

