# Supplementary material for: Precision nicotine metabolism-informed care for smoking cessation in Crohn’s disease: A pilot study
Source: PLoS One. 2020 Mar 26;15(3):e0230656. doi: 10.1371/journal.pone.0230656 (PMC7098646; doi:10.1371/journal.pone.0230656)
Supplement: S1 Table — (DOCX) [file pone.0230656.s001.docx]

| **Contraindications to Varenicline** |
| --- |
| History of seizures/ current condition/medication that lowers seizure threshold |
| Severe renal impairment (eGFR <30) |
| Pregnancy or breastfeeding |
| Prior hypersensitivity or adverse reaction |
| **Contraindications to Bupropion** |
| History of seizures/ current condition/medication that lowers seizure threshold |
| History of mania |
| Severe hepatic impairment |
| Use of monoamine oxidase inhibitor (MAOI) in prior 2 weeks |
| Pregnancy or breastfeeding |
| Prior hypersensitivity or adverse reaction |
| **Contraindications to Nicotine Replacement Therapy** |
| Recent (less than or equal to 2 weeks) myocardial infarction without revascularization, or unstable angina pectoris |
| Serious arrhythmias |
| Pregnancy or breastfeeding |
| Prior hypersensitivity or adverse reaction |

**Supplemental Table 1**. Contraindications to Smoking Pharmacotherapy Used for Study Eligibility
