# Supplementary material for: Precision nicotine metabolism-informed care for smoking cessation in Crohn’s disease: A pilot study
Source: PLoS One. 2020 Mar 26;15(3):e0230656. doi: 10.1371/journal.pone.0230656 (PMC7098646; doi:10.1371/journal.pone.0230656)
Supplement: S1 Fig — NMR was calculated as the ratio of 3-hydroxycotinine to cotinine as measured in serum. (DOCX) [file pone.0230656.s003.docx]

**S2 Fig. Distribution of NMR Values for the Entire Cohort of Participants.** NMR was calculated as the ratio of 3-hydroxycotinine to cotinine as measured in serum.
