# Supplementary material for: Precision nicotine metabolism-informed care for smoking cessation in Crohn’s disease: A pilot study
Source: PLoS One. 2020 Mar 26;15(3):e0230656. doi: 10.1371/journal.pone.0230656 (PMC7098646; doi:10.1371/journal.pone.0230656)
Supplement: S1 Protocol — (DOCX) [file pone.0230656.s004.docx]

**Implementation of Personalized Smoking Cessation Treatment into Inflammatory Bowel Disease Clinical Care: Pilot and Feasibility**

Elizabeth Scoville MD^1^

Quinn Wells MD, PharmD^2^

Matthew Freiberg MD^2^

Vanessa Gatskie MPH

Hilary Tindle MD, MPH^3^

Dawn Beaulieu MD^1^

**^1^Department of Medicine, Division of Gastroenterology**

**^2^Department of Medicine, Division of Cardiology**

**^3^Department of Medicine, Division of General Internal Medicine**

Table of Contents:

1. **Background**
2. **Rationale and Specific Aims**
3. **Animal Studies and Previous Human Studies**
4. **Inclusion/Exclusion Criteria**
5. **Enrollment/Randomization**
6. **Study Procedures**
7. **Risks of Investigational Agents/Devices (side effects)**
8. **Reporting of Adverse Events or Unanticipated Problems involving Risk to Participants or Others**
9. **Study Withdrawal/Discontinuation**
10. **Statistical Considerations**
11. **Privacy/Confidentiality Issues**
12. **Follow-up and Record Retention**
13. **References**
14. **Background**

The inflammatory bowel diseases, Crohn’s disease and ulcerative colitis are inflammatory conditions of the bowel characterized by chronic relapsing and remitting immune mediated inflammation. Tobacco use has been identified as an environmental risk factor for the onset of Crohn’s disease whereas in Ulcerative colitis smoking cessation has been correlated with onset of disease.[^1^](#_ENREF_1) In Crohn’s disease patients who smoke have more frequent relapses [^2-4^](#_ENREF_2), suffer more complications [^5^](#_ENREF_5), and require surgery more often[^6^](#_ENREF_6) than those who do not smoke. Patients who smoke also require higher levels of immunosuppressive therapy to control their disease than those who do not smoke[^7^](#_ENREF_7).

In addition to its adverse effects in Crohn’s disease, tobacco smoking is the leading preventable cause of disease and death in the US and the world. In all smokers, most quit attempts fail despite using FDA-approved pharmacotherapy.

Nicotine mediates its addictive effects in the brain, where it binds to CNS receptors, including nicotinic acetylcholine receptors (nAChRs).[^8^](#_ENREF_8) Nicotine undergoes rapid (half-life ~two hours) and extensive hepatic metabolism to cotinine, primarily by the enzyme CYP2A6.[^9^](#_ENREF_9) Cotinine, in turn, is further metabolized to 3-hydroxycotinine exclusively by hepatic CYP2A6.[^8^](#_ENREF_8) Genetic variation in pathways related to nicotine metabolism, primarily *CYP2A6*, have been associated with smoking related traits, including cigarette consumption,[^10^](#_ENREF_10)^,^ [^11^](#_ENREF_11) and response to treatment.[^12^](#_ENREF_12)

The ratio of 3-hydroxycotinine to cotinine, known as the nicotine metabolite ratio, or NMR, is a genetically- informed biomarker reflecting CYP2A6 activity and the rate of nicotine metabolism.[^13^](#_ENREF_13) A recent randomized control trial in *Lancet Respiratory Medicine* found that the NMR can be used to individualize treatment for smokers.[^14^](#_ENREF_14) Specifically, subjects who were identified as “fast” metabolizers (as opposed to “slow” metabolizers) demonstrated a two-fold improvement in cessation rates when treated with varenicline compared to nicotine replacement therapy (NRT). As a genetically-informed biomarker, NMR has been suggested as the tool of choice because it is inexpensive and has a relatively quick turnaround, lending feasibility to its role in guiding initial choice of pharmacotherapy. However, it is currently unknown whether a nicotine metabolism-guided approach utilizing NMR would be effective in a real world clinical environment targeting a population with a smoking related illness.

1. **Rationale and Specific Aims**

Without the use of proven quit aids, rates of smoking cessation are exceedingly low, with only 5-10% of smokers able to quit for 6 months or more.[^15^](#_ENREF_15) Approved drugs, including bupropion, NRT (i.e., patch, gum, lozenge, nasal spray, and inhalers), and varenicline, have each been shown to dramatically improve quit rates compared to placebo,[^16-18^](#_ENREF_16) and **current guidelines do not preferentially recommend any agent as first line therapy**. While this standard guideline-based approach does result in a 1.5 to 3-fold increase in success compared to placebo, the absolute rates of smoking cessation remain low. At one year, a quarter or less of smokers who use proven behavior therapy and pharmacotherapy remain abstinent.[^19^](#_ENREF_19) Despite the added importance of disease control, the rates of smoking cessation in inflammatory bowel disease patients are comparable to the general population. One small study of Crohn’s disease patients showed only 11% of motivated patients quit smoking in 6 months without intervention.[^20^](#_ENREF_20) A precision medicine approach utilizing NMR to tailor pharmacologic treatment is promising but unproven to increase quit rates and reduce smoking-related diseases.

**Our overarching hypothesis is that tailored smoking cessation therapy, guided by NMR, results in higher rates of tobacco cessation compared to usual care.** However, there are important challenges to be addressed prior to conducting a full-scale randomized controlled trial (RCT) comparing tailored treatment to usual care. We must establish that sufficient numbers of subjects can be enrolled; procedures for specimen collection, processing, and data analysis must be in place; and patient attitudes should be determined and considered in the study design.

The goal of this pilot project is **to create infrastructure, establish feasibility, and provide necessary preliminary data to design and conduct a prospective randomized trial comparing tailored smoking cessation treatment to usual care.** To accomplish this, we will enroll up to 100 outpatient smokers from the Vanderbilt Inflammatory Bowel Disease Clinic. After consent, subjects will complete questionnaires, provide biologic samples, and have nicotine metabolic status determined (nicotine metabolic ratio (NMR)). Subjects will be assigned to either: 1) usual care for smoking cessation or 2) personalized smoking cessation care which incorporates the NMR. Biochemically confirmed abstinence (by exhaled carbon monoxide, using a threshold of < 10 parts per million (ppm)) will be determined at the six month follow up.[^21^](#_ENREF_21) For deliverables, we will: 1) demonstrate capacity to conduct large-scale personalized smoking cessation trials in the clinical environment, and 2) assess patient attitudes regarding personalized smoking treatment.

**Aim 1. Develop infrastructure and procedures necessary to conduct a prospective randomized trial comparing tailored smoking cessation treatment to usual care.** We hypothesize that we will successfully: 1) enroll outpatient adult daily smokers; 2) collect, process, and store relevant biologic samples; 3) conduct follow-up assessments and accurately ascertain pertinent smoking cessation and patient reported outcome measures.

**Aim 2. Assess patient attitudes regarding personalized smoking therapy including: 1) interest in receiving personalized information, and 2) willingness to guide therapy based on personalized data.**

We hypothesize that a majority (>50%) of subjects will: 1) favorably view receiving personalized smoking information, and 2) express willingness to guide therapy based on personalized data.

1. **Animal Studies and Previous Human Studies**

Our group has not performed prior animal or human studies related to NMR. However, a recently published RCT in humans reported NMR can be used to guide smoking cessation therapy.[^14^](#_ENREF_14)

1. **Inclusion/Exclusion Criteria**

Inclusion criteria: Eligible subjects include adult (18 years of age or older) daily smokers who are seen in Vanderbilt IBD outpatient clinic. Current smoking is defined as smoking, on average, >5 cigarettes per day (on the days they smoked) in the month prior to enrollment. Potential subjects must agree to participate and be willing to accept a medication prescription for tobacco cessation. Subjects may initially be using other types of tobacco products but must be willing to give up all forms of tobacco, including electronic cigarettes (e-cigs), to be eligible for the study. Subjects must be eligible (i.e., no medical contraindications and willing to take) to receive at least two out of three FDA-approved smoking cessation medications (bupropion, varenicline, and NRT). The requirement of eligibility to take at least 2 of 3 FDA approved medications is necessary for this study so that individuals can take advantage of the NMR-driven guidance of a nicotine medication (i.e., NRT for slow metabolizers) vs. a non-nicotine medication (i.e., varenicline or bupropion for fast/normal metabolizers).

Exclusion criteria: In addition to medication contraindications, subjects will be excluded for inability to give informed consent or participate due to serious psychiatric (e.g., psychosis, schizophrenia, hospitalization for psychiatric condition in the past year) or cognitive disorder (e.g., dementia, severe mental retardation); receiving palliative or hospice care; pregnant or breastfeeding; no access to a telephone or inability to communicate by telephone; unable to speak and read English; use of smoking cessation medication in the 7 days prior to enrollment. Because levels of nicotine metabolites fall below detection over time we will exclude patients who have been completely abstinent from cigarettes for >3 days.

In order to maximize participant safety, eligibility for medications will be reviewed by three separate members of the study team. The study staff will conduct initial screening at the time of enrollment. Between enrollment and intervention phone call (~1 week) medication eligibility will be reviewed for each participant by the study co-investigator Dr. Scoville (or Dr. Beaulieu if not available) using a detailed chart review. Finally, at the time of intervention phone call, medication eligibility will again be confirmed by the study counselor, who is a nurse (RN).

1. **Enrollment/Randomization**

| Table 1 | |
| --- | --- |
| **Demographics of VUMC Outpatient IBD Clinic Active Smokers from October 2015-January 2016 (n=118)** | |
| Disease |  |
| Crohn's Disease, n (%) | 105 (88.9) |
| Ulcerative Colitis, n (%) | 12 (10.2) |
| Indeterminate Colitis, n (%) | 1 (0.8) |
| Gender |  |
| Male, n (%) | 58 (49.2) |
| Female, n (%) | 60 (50.8) |
| Age, median, yrs | 42 |

As part of an ongoing cohort (IRB #15110) we have identified approximately 118 patients who are routinely followed in the IBD clinic who are actively smoking. In addition, we will utilize existing infrastructure in the Vanderbilt IBD clinic to identify additional smokers. First, study staff will pre-screen scheduled patients in the EHR to identify smokers. Additionally, patients are routinely asked about their smoking status during every visit to the Vanderbilt IBD clinic during intake. Identified smokers will be asked by their providers or other members of the clinical team if they are interested in learning more about participation in smoking related research. Only those patients who express interest will be approached by the study staff for eligibility screening, consent, and possible enrollment. The study staff will screen for eligibility and, if appropriate, discuss the informed consent document with the prospective study participant. After agreeing to consent, the participant will complete a standardized questionnaire assessing his or her attitudes regarding personalized smoking cessation therapy. Participants will be randomized in blocks of 10, stratified based on self-reported baseline cigarettes per day.

1. **Study Procedures**

Baseline Data Collection

Upon enrollment patients will be given an initial survey detailing smoking history, smoking related traits (e.g. smoking intensity, measures of addiction, prior quit attempts, use of other tobacco products), and attitudes regarding personalized medicine. Participants will be compensated $10 for their completion of the survey. From chart review, we will collect baseline information regarding inflammatory bowel disease from review of the medical record (e.g type and duration of disease, disease activity, health related quality of life (SIBDQ) score, PHQ9, prior surgeries, and medications).

Biospecimen collection, processing, and storage

To minimize risk, a total of ~10 mL (well below the accepted cutoff of 50 mL for “minimal risk”) of whole blood will be collected into 1 EDTA coated tube (lavender top vacutainers), labeled appropriately, placed on ice. The sample will be transferred to the VUMC reference laboratory for measurement of nicotine, cotinine, 3-OH-cotinine. The VUMC laboratory currently contracts with ARUP national reference laboratory, which conducts this assay using quantitative high performance liquid chromatography-tandem mass spectrometry using CLIA approved protocols and quality control. Results are reported within 2-5 days from sample collection. (<http://ltd.aruplab.com/tests/pub/0092361)>

Nicotine metabolite ratio (NMR) measurement

As described above, subject samples will be processed through ARUP Laboratory as they would for standard clinical care. Results are available within 2-5 days from the time of blood draw.

Slow metabolizers will be defined by a NMR <0.31 and fast metabolizers will be defined by a NMR ≥ 0.31.[^14^](#_ENREF_14) Once available, subject NMR values will be entered into the study’s secure REDCap database by study personnel.

Study Intervention, medication provision and blinding

Medication Costs

All medications accepted by participants at the initial intervention phone call will be provided to participants free of charge. Subjects in the usual care arm who are still smoking at 6 months will have an opportunity to receive a prescription for smoking cessation therapy at the end of study (see below). Participants who wish to receive prescriptions at that point will be responsible for the cost of medications.

Usual Care Group

All subjects, including those assigned to usual care, will receive phone calls from clinical personnel within 1 month of enrollment. In order to minimize inconvenience, subjects will be asked their preferred times to be called at the time of enrollment. With the aid of a written script, staff will provide participants a verbal description of smoking cessation medications they are eligible to take (as determined by study physician), including efficacy and common side effects. If subjects would like to take a smoking cessation medication, they will be allowed to choose from those they are medically able to receive, and the study physician will call in a prescription to an outpatient pharmacy (with which arrangements have been made to have medication costs billed to the study rather than the subject). Regardless of whether subjects choose to receive a prescription for a medication, they will be offered referral to their state Tobacco Quit Line for smoking cessation via an online portal. This portal will track the patient’s progress through the quit line process and these data may be collected for research purposes. Verbal instructions will be provided to participants given prescriptions to contact their primary provider and the research study staff (during regular business hours) in case of adverse effects of a medication. They will also be instructed to call 911 if they believe they are having a medical emergency.

Personalized Care Group

All subjects, including those assigned to personalized care, will receive phone calls from clinical personnel within 1 month of enrollment. In order to minimize inconvenience, subjects will be asked their preferred times to be called at the time of enrollment. Staff contacting subjects in the personalized care group will have access to NMR values when they become available in the study’s secure REDCap database. With the aid of a written script, staff will provide participants a **personalized** (based on NMR) verbal description of smoking cessation medications they are eligible to take (as determined by study physician), including efficacy and common side effects. If subjects would like to take a smoking cessation medication, they will be allowed to choose from those they are medically able to receive, and the study physician will call a prescription into an outpatient pharmacy (with which arrangements have been made to have medication costs billed to the study rather than the subject). Regardless of whether subjects choose to receive a prescription for a medication, they will be offered referral to their state Tobacco Quit Line for smoking cessation counseling via an online portal. This portal will track the patient’s progress through the quit line process and these data may be collected for research purposes. Verbal instructions will be provided to participants given prescriptions to contact their primary provider and the research study staff (during regular business hours) in case of adverse effects of a medication. They will also be instructed to call 911 if they believe they are having a medical emergency.

Follow up phone calls

Study RN’s will call subjects at approximately 1 month post-intervention phone call and 3 and 6 months post-pharmacotherapy start date to assess smoking behavior, self-reported adherence, and side effects including neuropsychiatric side effects. Study personnel will make up to 10 calls per follow up at different times of day and days of the week and call subjects’ alternate contacts when needed. Participants will receive $10 for each phone call that they complete.

Single arm crossover for subjects in Usual Care Group

Subjects in the Usual Care Group who are still smoking at 6 month follow up will receive an additional intervention from study staff. With the aid of a written script, staff will provide participants a **personalized** (based on NMR) verbal description of smoking cessation medications they are eligible to take (as determined by study physician), including efficacy and common side effects. If subjects would like to take a smoking cessation medication, they will be allowed to choose from those they are medically able to receive, and a prescription will be called into an outpatient pharmacy by the study physician. The intent of this design is to assess the impact of a personalized message on medication uptake in participants who failed to quit with the usual care treatment. The cost of medications will be the responsibility of participants who chose to have prescriptions filled. Participants provided prescriptions will be called by study staff in approximately 2 weeks to assess whether they have filled their prescriptions and are taking the medication.

EHR data collection

Prospective participants who are identified smokers in the Vanderbilt IBD clinic will have their EHR examined by study staff to determine eligibility, and EHR data collected in the screening form will be retained for future analysis. For enrolled participants, research staff will review the participant’s EHR to collect relevant clinical data. At 3 and 6 months we will collect clinical information regarding inflammatory bowel disease characteristics from review of the medical record (e.g disease activity, health related quality of life (SIBDQ) score, PHQ-9 score, hospitalizations, surgeries, and medications). Staff may review participant records for up to 5 years after enrollment.

Outcome assessment

Subject outcomes will be assessed by phone approximately 1 month post-intervention phone call and 3 and 6 months post-pharmacotherapy start date. For those participants who have not started a medication by 1-month, 3 and 6 months follow-up will be based on the post-intervention phone call. At follow up, a research assistant will complete interviews assessing smoking behavior, self-reported side effects, and measures of patient engagement and attitudes toward precision medicine. At approximately 6 months, subject outcomes may be assessed in person. Subjects self-reporting abstinence may have smoking status confirmed biochemically using end expired carbon monoxide. Biochemically validated abstinence will be defined as end expired carbon monoxide less than 10 ppm. Participants will receive $10 for each of these completed calls and an additional $10 if they participate in an end-expired carbon monoxide test at 6 months. This will be sent by mail in the form of a check. Participants in the single arm crossover group (i.e., those in the Usual Care group who were still smoking at 6 months) who accepted medication prescriptions will receive phone calls approximately 2 weeks after the second personalized intervention to assess whether they have filled the prescription. The most any one participant can be compensated (completing all surveys and biochemical validation) is $60.00 over the course of approximately 6 months. See Table 2 for a breakdown of follow-up and outcome measures.

Table 2: Follow-up and Outcome Measures Summary

|  | **Baseline** | **1 mo*** | **3 mo**** | **6 mo**** | **Final follow up^+^** |
| --- | --- | --- | --- | --- | --- |
| **Outcomes** |  |  |  |  |  |
| Demographics | X |  |  |  |  |
| Smoking behavior | X | X | X | X | X |
| Side effect screening | X | X | X |  |  |
| Attitudes & Beliefs | X |  |  | X |  |
| Patient satisfaction |  |  |  | X |  |
| Biochemical validation of cessation |  |  |  | X |  |
| Medication use |  | X | X |  | X |

* Relative to Intervention phone call.

** Relative to medication start date

^+^ 2 weeks after 6 month follow up

1. **Risks**

Risks of blood draw: Pain, redness, soreness, bruising, or infection may occur at the needle stick site. Rarely, some patients faint. We will protect against phlebotomy-related risk by collecting samples at the time of clinical sample collection when possible.

Expired Carbon Monoxide Test: There are no known serious adverse effects from this test. Sometimes people feel mildly short of breath or cough during the test.

Breach of Confidentiality: Medical information will be stored in a secure database. However, it is possible this database could be corrupted. Participant data will be collected using password-protected and encrypted computers, electronic medical records, and electronic participant tracking spreadsheets stored on a secure server. To prevent the loss of data, all electronic information is stored within the VUMC firewall and is password-protected. Only study staff will have access to the study data. Data quality (including visits completed during intervention window, data missingness, and recruitment rates) will be monitored monthly by the database manager and systematic data problems will be reported to the PI (Dr. Beaulieu).

Adverse drugs reactions: Subjects in this study will receive one of three FDA-approved smoking cessation therapies (NRT, varenicline, or bupropion) and will have the potential to experience drug-related side effects. Nicotine replacement therapy is generally very safe (it is sold over the counter). However, possible side effects include skin irritation or itching (patch), dizziness, headache, nausea, and rapid heartbeat. Common side effects of varenicline include nausea, sleep disturbance and vivid dreams, gastrointestinal symptoms, and vomiting. Very rarely, patients, particularly those with unstable psychiatric conditions, may experience symptoms such as behavioral changes, agitation, depressed mood, and suicidal behavior during varenicline treatment. Common side effects of bupropion include dry mouth, irritability, and difficulty sleeping. A large trial of over 8000 patients was presented at the Society for Research on Nicotine and Tobacco in Chicago in March, 2016, and did not demonstrate any difference in neuropsychiatric symptoms (including suicidal ideation and suicidal behavior) between patients on varenicline, bupropion, or nicotine replacement therapy (EAGLES; ClinicalTrials.gov Identifier: NCT01456936). Very rarely, patients can have seizures, especially in those with a history of seizures. To protect against risk from pharmacotherapy usage, this study will only use FDA-approved smoking cessation medications and tobacco treatment providers will screen patients to identify contraindications to NRT, bupropion or varenicline. Written and verbal instructions will be provided to patients given medication to contact their primary physician and the research PI, Dawn Beaulieu, in case of adverse effects of a medication. Additionally, RAs will call subjects at regular intervals (as described above) to monitor for serious side effects including neuropsychiatric side effects. Study personnel will make up to 10 calls per week at different times of day and days of the week and call subjects’ alternate contacts when needed. Ultimately, the PI (Dr. Beaulieu) will review any serious adverse events and report them appropriately to the IRB.

1. **Reporting of Adverse Events or Unanticipated Problems Involving Risk to Participants or Others**

Any adverse events will be reported to the IRB according to IRB policies and procedures.

1. **Study Withdrawal/Discontinuation**

Individuals who wish to withdraw from the study may do so at any time by contacting the Principal Investigator (Dr. Beaulieu) in writing without penalization or change to their medical care. Any data collected prior to withdrawing from the study will be analyzed as enrolled unless requested for full withdrawal by the participant.

1. **Statistical Considerations**

This pilot project is intended to create infrastructure, establish feasibility, and provide necessary preliminary data to design and conduct a prospective RCT comparing tailored smoking cessation treatment to usual care. As such, we are not powered to detect differences between treatment groups in regards to primary or secondary outcomes. However, we will conduct descriptive and exploratory analyses of collected data as well as assess for between-group differences.

**Aim 1. Develop infrastructure and procedures necessary to conduct a prospective randomized trial comparing tailored smoking cessation treatment to usual care.**

Hypothesis: We hypothesize that we will successfully: 1) enroll adult daily smokers from an outpatient clinic; 2) collect, process, store, and analyze relevant biologic samples; and 3) conduct follow-up assessments and accurately ascertain pertinent smoking cessation and patient care outcome measures.

Primary analysis: As a primary analysis, for each group we will calculate descriptive statistics for relevant demographic and clinical parameters, rates of enrollment and loss to follow-up, smoking cessation, and side effects. These data will be critical to inform future prospective studies.

**Aim 2. Assess patient attitudes regarding personalized smoking therapy including: 1) interest in receiving personalized information, and 2) willingness to guide therapy based on personalized data.**

Hypothesis: A majority (>50%) of subjects will: 1) favorably view receiving personalized smoking information; and 2) express willingness to guide therapy based on personalized data.

Primary analysis: As a primary analysis, we will calculate the proportion of subjects that provided favorable responses (4 or 5 on Likert Scale) for questions addressing: 1) willingness to receive patient-specific smoking data; and 2) willingness to use these data to guide therapy, as reflected by the % who fill the prescription that is recommended by the NMR test results. We will also calculate rates of self-reported adherence, use of the quit line, and cessation at each follow up.

1. **Privacy/Confidentiality Issues**

Data will be placed into a password-protected, web-based database by study personnel. Only the research staff, the PI and co-investigators will have access to this data. A unique identification number will be used to protect the confidentiality of the study participants.

Protected Health Information (PHI) will be used in this study. The investigators will comply with the patient privacy guidelines of Vanderbilt University Medical Center and the rules outlined by the Health Insurance Portability and Accountability Act (HIPAA).

All research samples obtained for this study will be assigned a unique number. The key linking this unique number to PHI will be kept in a locked file. Only the investigator, co-investigators and research personnel will have access to the key and information that identifies participants as being in this study. Digital records will be stored on password-protected computers/servers. Digital data files will be coded so that the participant name or other such identifiers are not in the filename.

1. **Follow-up and Record Retention**

Subjects will be followed up through approximately 6 months after intervention phone call or post-pharmacotherapy start date, if applicable (as outlined above in Study Procedure). Records will be kept indefinitely but will be destroyed if no longer needed.

1. **References**

1. Mahid SS, Minor KS, Soto RE, Hornung CA, Galandiuk S. Smoking and inflammatory bowel disease: A meta-analysis. *Mayo Clinic Proceedings*. 2006;81:1462-1471

2. Duffy LC, Zielezny MA, Marshall JR, Weiser MM, Byers TE, Phillips JF, Ogra PL, Graham S. Cigarette smoking and risk of clinical relapse in patients with crohn's disease. *American journal of preventive medicine*. 1990;6:161-166

3. Russel MG, Nieman FH, Bergers JM, Stockbrugger RW. Cigarette smoking and quality of life in patients with inflammatory bowel disease. South limburg ibd study group. *European journal of gastroenterology & hepatology*. 1996;8:1075-1081

4. Breuer-Katschinski BD, Hollander N, Goebell H. Effect of cigarette smoking on the course of crohn's disease. *European journal of gastroenterology & hepatology*. 1996;8:225-228

5. Lindberg E, Järnerot G, Huitfeldt B. Smoking in crohn's disease: Effect on localisation and clinical course. *Gut*. 1992;33:779-782

6. Cottone M, Rosselli M, Orlando A, Oliva L, Puleo A, Cappello M, Traina M, Tonelli F, Pagliaro L. Smoking habits and recurrence in crohn's disease. *GASTROENTEROLOGY-BALTIMORE THEN PHILADELPHIA-*. 1994;106:643-643

7. Cosnes J, Carbonnel F, Carrat F, Beaugerie L, Cattan S, Gendre J. Effects of current and former cigarette smoking on the clinical course of crohn's disease. *Alimentary Pharmacology and Therapeutics*. 1999;13:1403-1412

8. Benowitz NL. Pharmacology of nicotine: Addiction, smoking-induced disease, and therapeutics. *Annual review of pharmacology and toxicology*. 2009;49:57

9. Hukkanen J, Jacob P, Benowitz NL. Metabolism and disposition kinetics of nicotine. *Pharmacological reviews*. 2005;57:79-115

10. Fujieda M, Yamazaki H, Saito T, Kiyotani K, Gyamfi MA, Sakurai M, Dosaka-Akita H, Sawamura Y, Yokota J, Kunitoh H. Evaluation of cyp2a6 genetic polymorphisms as determinants of smoking behavior and tobacco-related lung cancer risk in male japanese smokers. *Carcinogenesis*. 2004;25:2451-2458

11. Thorgeirsson TE, Gudbjartsson DF, Surakka I, Vink JM, Amin N, Geller F, Sulem P, Rafnar T, Esko T, Walter S. Sequence variants at chrnb3-chrna6 and cyp2a6 affect smoking behavior. *Nature genetics*. 2010;42:448-453

12. Chen LS, Bloom AJ, Baker TB, Smith SS, Piper ME, Martinez M, Saccone N, Hatsukami D, Goate A, Bierut L. Pharmacotherapy effects on smoking cessation vary with nicotine metabolism gene (cyp2a6). *Addiction*. 2014;109:128-137

13. Dempsey D, Tutka P, Jacob P, Allen F, Schoedel K, Tyndale RF, Benowitz NL. Nicotine metabolite ratio as an index of cytochrome p450 2a6 metabolic activity. *Clinical Pharmacology & Therapeutics*. 2004;76:64-72

14. Lerman C, Schnoll RA, Hawk LW, Cinciripini P, George TP, Wileyto EP, Swan GE, l Benowitz N, Heitjan DF, Tyndale RF. Use of the nicotine metabolite ratio as a genetically informed biomarker of response to nicotine patch or varenicline for smoking cessation: A randomised, double-blind placebo-controlled trial. *The lancet Respiratory medicine*. 2015;3:131-138

15. Messer K, Trinidad DR, Al-Delaimy WK, Pierce JP. Smoking cessation rates in the united states: A comparison of young adult and older smokers. *American journal of public health*. 2008;98:317-322

16. Gonzales D, Rennard SI, Nides M, Oncken C, Azoulay S, Billing CB, Watsky EJ, Gong J, Williams KE, Reeves KR. Varenicline, an α4β2 nicotinic acetylcholine receptor partial agonist, vs sustained-release bupropion and placebo for smoking cessation: A randomized controlled trial. *Jama*. 2006;296:47-55

17. Jorenby D, Hays J, Rigotti N. A randomized controlled trial comparing the efficacy of varenicline, a novel α4β2 nicotinic acetylcholine receptor partial agonist, to bupropion and to placebo for smoking cessation. *JAMA*. 2006;296:56-63

18. Hughes JR, Stead LF, Lancaster T. Antidepressants for smoking cessation. *Cochrane Database Syst Rev*. 2014;1

19. Bauld L, Bell K, McCullough L, Richardson L, Greaves L. The effectiveness of nhs smoking cessation services: A systematic review. *Journal of Public Health*. 2010;32:71-82

20. Leung Y, Kaplan GG, Rioux KP, Hubbard J, Kamhawi S, Stasiak L, Cohen RD, Devlin SM, Panaccione R, Hanauer SB. Assessment of variables associated with smoking cessation in crohn’s disease. *Digestive diseases and sciences*. 2012;57:1026-1032

21. Dorfman S. Treating tobacco use and dependence: 2008 update us public health service clinical practice guideline executive summary. *Respiratory Care*. 2008;53:1217-1222
